# Heat shock proteins and viral infection

**DOI:** 10.3389/fimmu.2022.947789

**Published:** 2022-08-05

**Authors:** Xizhen Zhang, Wei Yu

**Affiliations:** ^1^ Institute of Biochemistry, College of Life Sciences and Medicine, Zhejiang Sci-Tech University, Hangzhou, China; ^2^ Zhejiang Provincial Key Laboratory of Silkworm Bioreactor and Biomedicine, Hangzhou, China

**Keywords:** HSPs, viral infection, chaperones, protein folding, immunological pathways

## Abstract

Heat shock proteins (HSPs) are a kind of proteins which mostly found in bacterial, plant and animal cells, in which they are involved in the monitoring and regulation of cellular life activities. HSPs protect other proteins under environmental and cellular stress by regulating protein folding and supporting the correctly folded structure of proteins as chaperones. During viral infection, some HSPs can have an antiviral effect by inhibiting viral proliferation through interaction and activating immune pathways to protect the host cell. However, although the biological function of HSPs is to maintain the homeostasis of cells, some HSPs will also be hijacked by viruses to help their invasion, replication, and maturation, thereby increasing the chances of viral survival in unfavorable conditions inside the host cell. In this review, we summarize the roles of the heat shock protein family in various stages of viral infection and the potential uses of these proteins in antiviral therapy.

## Introduction

Heat shock proteins (HSPs) were first discovered in the salivary glands of flies, where they are expressed under heat shock conditions. HSPs have a wide range of molecular weights from approximately 10 to 100 kDa and can be classified into different groups according to their molecular weight, including small heat shock proteins (sHSPs), HSP40, HSP60, HSP70, HSP90 and large heat shock proteins (1).

The sHSPs, most of which are heat-inducible, have a wide range of molecular weights from 12-43 kDa and are widely distributed in a variety of tissues. The ability to prevent the aggregation of proteins and polypeptides is the most important function of many sHSPs (2). Depending on the status of client proteins, sHSPs exert different molecular chaperone functions (3–5). HSP40, HSP60, HSP70 and HSP90 are well-studied heat shock proteins that often perform biological functions in cells as complexes. They are extensively involved in the lifecycle of proteins, including protein folding and refolding, transport, degradation, assembly, activity regulation, and translocation, as well as the depolymerization of protein aggregates. The heat shock protein family is also involved in many fundamental cellular processes including cell cycle control, cell survival, hormone signaling and response to cellular stress through the extensive regulation of intracellular proteins (6–11). Large HSPs, such as HSP100 and HSP110, contain a loop structure that gives them a high capacity of binding to polypeptide substrates or non-protein ligands such as pathogen-associated molecules (12). Both HSP100 and HSP110 have chaperone activity with HSP70, and they can regulate protein aggregation by forming HSP104-HSP70-HSP40 (13) and HSP110-HSP70-HSP40 (14) ternary complexes to maintain cellular homeostasis in a variety of cellular life activities.

The expression of HSPs is not only induced by heat or cold but is also responsive to a range of stressors including starvation (15), hypoxia (16), ultraviolet (UV) irradiation (17), exposure to heavy metals (18) and microbial infection (19). During viral infection, HSPs protect the host cells mainly by their chaperone functions. Small heat shock proteins are produced in large quantities in response to stress (20), partly activating immune signaling pathways (21), and partly assembling complexes to modulate apoptosis (22). The bigger members also assemble HSP complexes to fold host proteins correctly and refold aggregates of stress-denatured proteins (23). Importantly, some heat shock proteins are directly involved in the inhibition of viral replication and transcription (24).

Although the HSP family is a class of protective proteins, they can be hijacked by viruses to aid host cell invasion. Viruses lacking molecular chaperones can utilize the native HSPs of the host cell to help them invade cells and the nucleus (25), stabilize and regulate their own transcription and translation (26), assemble viral proteins, or alter the intracellular environment to promote viral proliferation (27, 28). The aim of this review is to collate relevant reports on the role of the HSP family in various phases of viral infection and pave the way for subsequent research on related treatments.

## Responses of HSPs under different stress conditions

Based on the function of chaperones, HSPs widely participate in biomolecular networks by binding to proteins of various functions in space and time (**Table 1**). Under normal conditions, HSPs play a role in the regulation of the cellular life cycle and functions, while under stress, HSPs are one of the main systems to be activated and regulate stress resistance, thereby enhancing viability. The main stressors that organisms face can be roughly divided into three categories, including physical, chemical and biological factors (19). Here, we briefly summarize the responses of HSPs in response to the most common stressors such as cold, anoxia and pathogenic microorganisms.

**Table 1:** Functions and distribution of heat shock proteins.

HSP	Intracellular Distributions	Functions
HSP100	Cytoplasm Nucleus	Dissociation, refolding, and re-solubilization of protein aggregates
HSP90	Cytoplasm Endoplasmic Reticulum (ER) Mitochondria Nucleus	Modification of kinases, steroid hormone receptors, and transcription factors
HSP70	Chloroplasts Cytoplasm Endoplasmic Reticulum (ER) Mitochondria Nucleus	Unfolds misfolded polypeptides Translocates unfolded polyproteins through membranes Dissociates protein complexes
HSP60	Chloroplasts Cytoplasm Mitochondria	Segregates unfolded polypeptide chains Promotes unfolding of misfolded polypeptides by both active and passive mechanisms
HSP40	Cytoplasm Nucleus	Folding, Degradation and Translocation of Proteins
HSP27 (sHSP)	Cytoplasm Nucleus	Maintain cytoskeletal protein stability Against apoptosis

**Figure 1:**
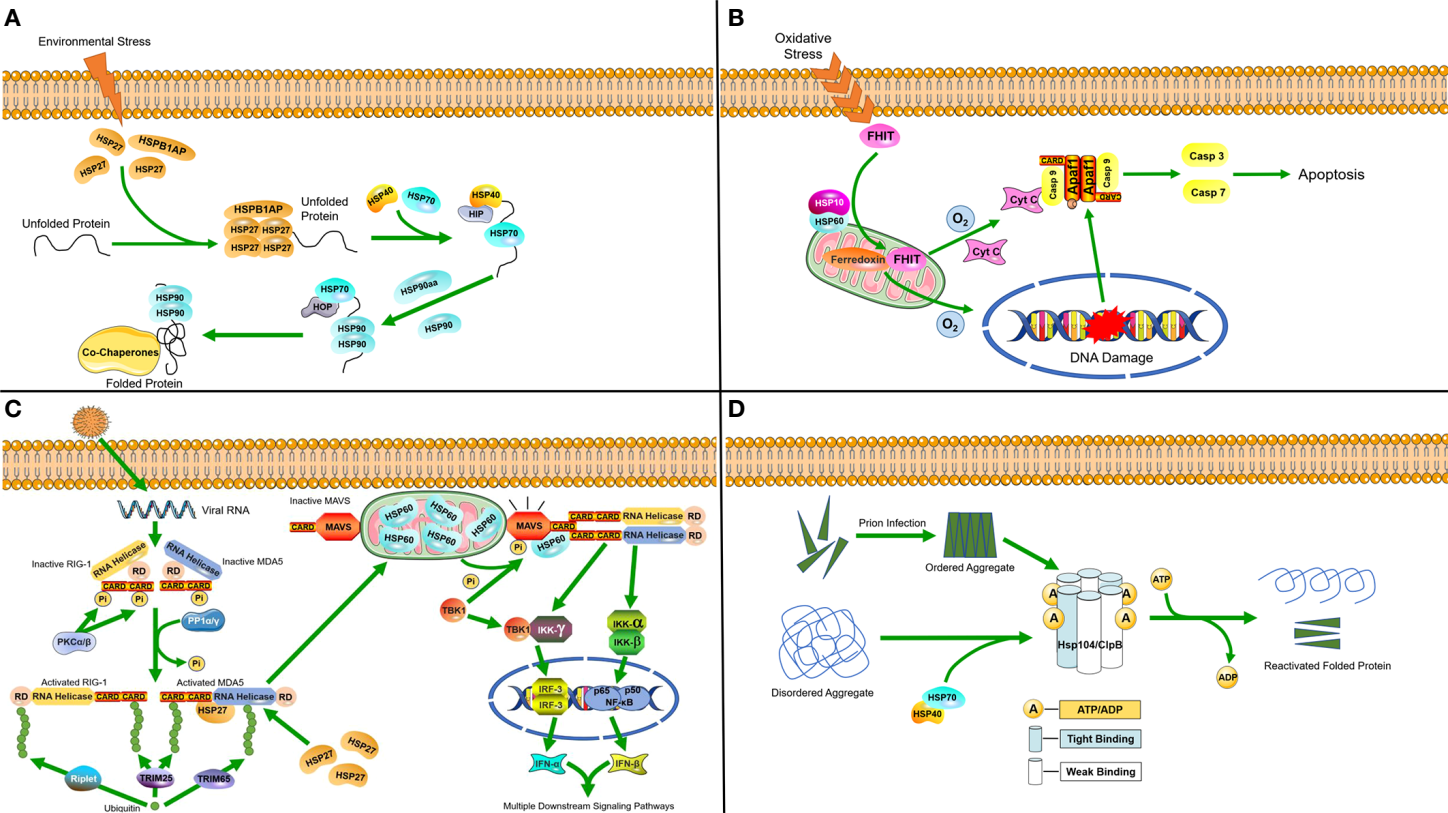
Molecular mechanisms of heat shock proteins induced under stress **(A)** Hsp27 binds to unfolded proteins that accumulate in the cytosol during stress, and then diverts unfolded proteins along the protein folding pathway, ultimately reaching HSP90. **(B)** In response to oxidative stress, the HSP60-10 complex helps to localize FHIT protein to the mitochondria, where it stabilizes ferredoxin reductase, leading to enhanced production of reactive oxygen species. This in turn triggers cytochrome c release and subsequent activation of the caspase cascade, ultimately causing apoptosis. **(C)** Following viral invasion, the RLR/MDA5 signaling pathway is activated. HSP27 can specifically stabilize MDA5 during expression to enhance the RLR/MDA5 signaling pathway. In mitochondria, HSP60 interacts with MAVS to increase MAVS-mediated IFN-β promoter activity and the transcriptional levels of IFN-β. Furthermore, it can upregulate MAVS-induced mRNA transcription of IFN-stimulated genes (ISGs). **(D)** Hsp104/ClpB complexes in host cells process disordered aggregates accumulated following cellular stress as well as ordered aggregates formed after prion infection with the help of the HSP70-40 partner system, dissociating them into component proteins and reactivating them.

In response to stressful environments, HSPs regulate transcription and translation by acting as accessory proteins. In a study on the cold adaptation of the Asiatic rice borer moth, *Chilo suppressalis (* 20), small HSPs (sHSPs) were found to act as the first line of cellular defense against protein unfolding caused by the environmental stress, since their protein depolymerization activity is independent of ATP (29). They are capable of binding a large range of non-native substrate proteins to form sHSP-substrate complexes that prevent irreversible aggregation (30). Four sHSP genes were found in the genome of *C. suppressalis*, three of which are highly induced in response to cold stress and associated with HSP Beta-1 (HSPB1)-related protein (HSPB1AP). HSP70 and HSP90 are then synergistically upregulated at the transcriptional level, which requires the participation of HSP40 (31) and a protein called HSP90 ATPase homolog activator (HSP90aa) (32). Afterwards, HSP70 and HSP90 cooperatively refold proteins (**Figure 1A**) (33, 34).

At normal levels of oxygen, HSP60 forms a complex with the pro-apoptotic factor BCL2-associated X (Bax) in the cytosol and inhibits its translocation into the mitochondria, thereby preventing apoptosis. However, when cells are faced with hypoxia, the formation of complexes will be reduced and release Bax for translocation into the mitochondria, which results in the release of cytochrome c as an apoptotic signal (35). The HSP60-10 complex responds to oxidative stress and induces apoptosis when cells are under the dual stress of hypoxia and DNA damage (**Figure 1B**) (36).

HSPs are also induced when the host cell is infected by pathogenic microorganisms. A study on porcine reproductive and respiratory syndrome virus (PRRSV) identified HSP60 as a novel antiviral protein that inhibits viral replication (37). PRRSV infection activates PP1α/γ to dephosphorylate the originally phosphorylated MDA5 and RIG-1, after which MDA5 and RIG-1 are activated through ubiquitination. MAVS is phosphorylated and activated by TBK1, after which it interacts with RIG-I or MDA5, which triggers formation of a signaling synapse resulting in the formation of the canonical IFN-β enhanceosome complex that promotes IFN-β transcription. The RLR/MDA5 (RIG-I like receptor/melanoma differentiation-related gene 5) signaling pathway promotes the production of type I interferon to active the downstream signaling pathways (38). Upon the activation of mitochondrial antiviral signaling proteins (MAVS), HSP60 from the mitochondria binds to the MAVS protein and increases the expression of IFN-β, which can inhibit viral replication (37, 39). A recent study also found that the chaperone HSP27 positively regulates the RLR/MDA5 signaling pathway, which is triggered by encephalomyocarditis virus (EMCV) by stabilizing the expression of MDA5 to inhibit viral replication (**Figure 1C**) (40)

As molecular chaperones, heat shock proteins function by binding to client proteins in response to cellular or organismal stress. Small heat shock proteins bind directly to the target protein to prevent it from unfolding (2) or to transfer it to a complex transfer it to a complex which is inclined to be formed by larger heat shock proteins for further folding or refolding (20, 23). In response to stress, heat shock proteins either bind directly to protect the target protein (20), or affect factors that regulate cellular activities such as apoptosis (35) and immune signaling pathways (38). HSPs are activated by a wide variety of cellular stresses to maintain cellular homeostasis. However, it is also this characteristic that makes HSPs an easy target for viruses to break through host defenses. In this review, we will focus on the main functions of HSPs in viral infection.

## Biological functions of HSPs in host cells during viral infection

There are a large number of studies reporting that HSPs are not only involved in antiviral responses, but also could be utilized by the virus to help cell entry, viral replication and virion assembly.

## Antiviral activity of HSPs

As a class of protective proteins, HSPs can inhibit viral proliferation by interacting with viral molecules and their related proteins. For example, HSC70/HSP90 has already been confirmed to be a driving force of the RNA-induced silencing complex (RISC) assembly pathway by providing ATP to load small RNA duplexes into argonaute protein, which can promote complex formation (24). Subsequently, RISC binds to viral mRNA, leading to the repression of viral translation. In the study of HPV, it was found that secreted HSP70 can effectively target dendritic cells with relevant antigens to enhance the antigen-specific immune response (41). The ClpB/HSP104 complex can disassemble disordered aggregates that accumulate due to cellular stress, as well as ordered aggregates formed by prions with the help of the HSP70 chaperone system, and reactivate their constituent proteins (**Figure 1D**) (23).

**Figure 2:**
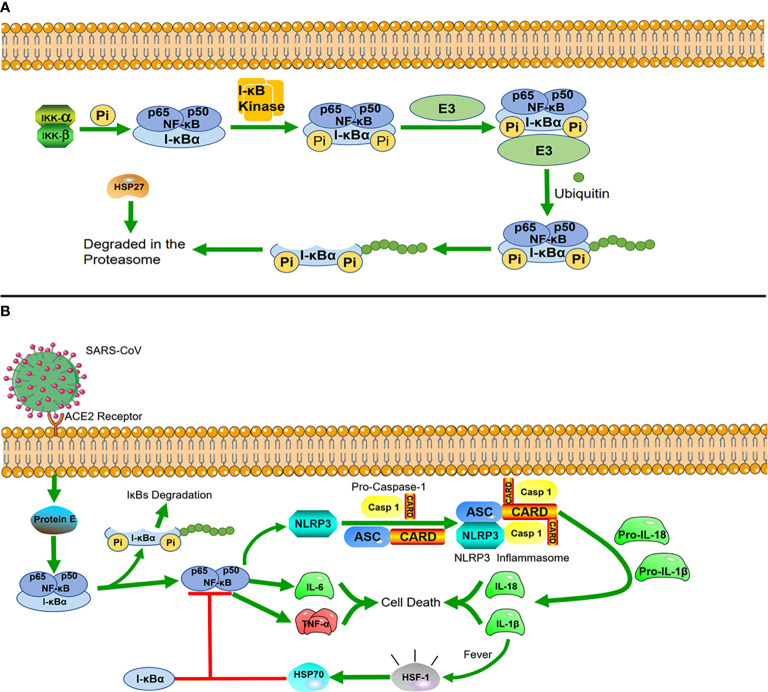
Heat shock proteins and immunological pathways **(A)** HSP27 regulates the NF-κB pathway. In the NF-κB signaling pathway, nuclear factor κB mainly exists as a heterodimer of p65 and P50, and I-κBα is a major inhibitor of NF-κB, which combines with them to form a complex in the resting state. The dimers are held inactive in the cytoplasm by their interaction with I-kBα proteins. I-κBα is phosphorylated when stimulated by external signals, and after phosphorylation, I-κBα proteins undergo ubiquitin-dependent degradation by the proteasome, after which NF-κB is translocated to the nucleus, where it acts as a transcription factor. The interaction of HSP27 with the 26S proteasome is necessary for the degradation of phosphorylated I-κBα, and overexpression of HSP27 enhances the proteasomal degradation of phosphorylated I-κBα. **(B)** HSP70 negatively regulates NLRP3 inflammatory vesicles. After SARS-CoV infects cells, the virus envelope E protein triggers the activation of the NF-κB inflammatory signaling cascade, which activates the NLRP3 inflammasome. Activation of NLRP3 induces the maturation of caspase-1, which in turn activates the secretion of interleukins IL-1β and IL-18. While IL-1β is an important factor in inducing a rise in core body temperature, HSP70, produced in response to the heat shock factor 1 (HSF-1), reduces the inflammatory response blocking NLRP3 and the articulator ASC to induce caspase-1 precursor maturation following a rise in body temperature.

HSP can also regulate immune signaling pathways to resist viral infections. As mentioned above, HSP60 regulates the RLR/MDA5 signaling pathway to influence cellular immunity. In addition, HSP40 was also found to bind to MDA5 in the MDA5-MAVS pathway to disrupt the formation of MDA5 multimers, resulting in the suppression of type I IFN induction and protecting host cells from damage caused by excessive inflammation triggered by viral infection (42). Many studies have found that the upregulation of HSP27 inhibits the replication of porcine epidemic diarrhea virus (PEDV) and red spotted grouper neuro necrosis virus (RGNNV). HSP27 significantly increases the phosphorylation of NF-κB as an upstream regulator, which in turn upregulates interferon promoter activity and activates downstream interferon-stimulated genes. Viruses have also developed counteracting strategies to significantly downregulate HSP27 expression (43, 44). In general, HSP27 interacts with many different viral proteins to regulate the activity of IFN-1 and NF-κB signaling pathways (**Figure 2A**) (21, 45).

During the two years of the COVID-19 epidemic, many studies on inflammation caused by coronavirus infection have been reported, in which we can also find new roles of HSPs. The evolutionarily conserved innate immune system is the first defense line against viral infection (46). The innate immune system is highly sensitive to stimuli, which rapidly recruit cells within minutes (neutrophils) to hours (monocytes/macrophages) to the site of injury. These rapid responses are orchestrated primarily by the expression of NF-κB, which drives inflammation during the early phase (47). There is a unique class of cytoplasmic receptors in the innate immune system called nucleotide-binding and oligomerization domain (NOD)-like receptors (NLRs), which constantly patrol for invading pathogens in the cytoplasm. At the heart of damaging inflammatory responses in many diseases is a multimolecular complex called the NOD-like receptor protein 3(NLRP3) inflammasome (48). In COVID-19, the viral envelope E protein triggers the activation of the NF-κB inflammatory signaling cascade and the interaction with inflammatory factors, such as tumor necrosis factor-alpha (TNF-α) and interleukin 6 (IL-6). These changes act as strong stimuli activating the cytosolic innate immune NLRP3 inflammasome. Once constituted, the NLRP3 inflammasome is secreted from the cells and can amplify the inflammatory response by activating the inflammasome and caspase-1 in neighboring cells. A recent study found that overexpression of HSP70 can inhibit the activation of the NLRP3 inflammasome, which in turn regulates the activation of caspase-1 (49)and the maturation of IL-1β (50) (**Figure 2B**). In related drug treatment studies, HSP90 inhibitors were found to block the initiation and activation of the NLRP3 inflammasome (51, 52).

Once the cells activate the inflammatory response, cyclooxygenase-2 (COX-2) is induced and starts producing proinflammatory arachidonic acid-derived prostaglandins (PGs) to promote the repair of inflammatory cells and tissues. Furthermore, PGs lead to an increase of the core body temperature (fever), which also triggers the heat shock response (HSR) (53). Under the influence of fever, structural changes in the plasma membrane directly activate heat shock factor 1 (HSF-1), whcih regulates the transcription of HSPs, expression of cytokines, and early response genes. The production of HSP70 in response to HSF-1 activation is correlated with complex formation between NF-κB and its inhibitor (I-κB) to prevent the translocation of NF-κB into the nucleus, which downregulates the acute inflammatory response (54). This avoids excessive protein damage or a cytokine storm induced by excessive inflammation (55).

## Viral binding and internalization

Attachment is the first crucial step in the initiation of viral infection. It depends on the interaction between the viral attachment proteins and cellular receptors, which are key determinants of viral host specialization and pathogenesis. As a family of chaperone proteins widely distributed in cells, HSPs have been found to act as receptors for a variety of viruses in recent studies (56–58), and they are mainly involved in the viral contact and cell invasion in two ways.

Firstly, they participate in the process of viral entry into cells through endocytosis mediated by endocytosin and clathrin. A large number of helper proteins involved in endocytosis mediated by clathrin are present in various cells, and the HSP family is also represented among them. The D isoform of heat shock cognate protein 70 (HSC70) was found to help Japanese encephalitis virus (JEV) penetrate C6/36 cells *via* clathrin-mediated endocytosis (58). Another important chaperone, HSP90, was also recently found to form a complex with red spotted grouper neuronecrosis virus (RGNNV) on the cell surface and independently lead to RGNNV internalization through the clathrin endocytosis pathway (59).

Similarly, HSPs can also directly bind to virions as receptors on the cell surface. In existing reports, HSP70 was found to be involved in the invasion of various viruses in C6/36 cells. For example, HSC70 is involved in the process of dengue virus (DENV) invasion of cells by interacting with the DENV receptor complex (60, 61), while HSC70 interacts with the VP5 subunit of rotavirus spike protein to help it enter cells through endocytosis (24). Similar to HSP70, HSP90 is also an important component of the dengue virus receptor complex. In the available literature, HSP90 was found to be utilized directly as a cell surface to regulate receptor-mediated endocytosis pathways by many viruses, such as infectious bursal disease virus (62), dengue virus (63) and Japanese encephalitis virus (64). HSP90AA1 is a subtype of the HSP90 family and it was found to be involved in the cell entry of influenza A virus (IAV). IAV was reported to initiate the entry process *via* multiple endocytic pathways mediated by the viral hemagglutinin (HA) glycoprotein (65). HSP90AA1 is distributed on the cell surface and can regulate the entry of IAV directly by interacting with viral hemagglutinin (HA) (64).

## Viral entry into the nucleus

Some viruses need to translocate viral molecules into the nucleus to interfere in the regulation of the cell’s internal environment or to advance replication of the viral own genome after invading a cell. HSPs are also involved in nuclear transport or the regulation of the intracellular environment to favor virion production, such as inducing tubulin acetylation to arrest the cell cycle (66) and so on. In IAV infection, HSP90 first exhibits downregulated acetylation levels along with enhanced nuclear transport to assist viral polymerase nuclear entry, after which the virus induces an upregulation of HSP90 acetylation levels, which indicates that HSPs play different roles at different phases of infection (67). Early in the IAV infection process, HSP40 (DnaJB1) can bind to the nucleoprotein (NP) of IAV with a nuclear localization signal and assists IAV viral ribonucleoprotein (vRNP) with nuclear trafficking through its interaction with nucleoproteins, which is also very important for viral protein entry (25). Similarly, HSP90 plays a role in enhancing the interaction between viral proteins and tubulin by binding to the acetylated α-tubulin to upregulate nuclear transport, which has been found in several viral infections, including mouse polyomavirus and herpes simplex virus 1 (68, 69).

## Viral replication, transcription and translation

HSPs not only assist in the nucleation of viral molecules, but are also intimately involved in the replication, transcription and translation of viruses, mainly in two ways. Since the HSP family is an important class of chaperones, they generally combine with virus-associated proteins to participate in their replication. Murine latency-associated nuclear antigen (mLANA) is a conserved protein of murine gammaherpesvirus 68 (MHV68) that is of great importance to latency maintenance and acute viral replication. In MHV68-infected 3T12 fibroblasts, mLANA directly interacts with HSC70 and recruits it to accumulate in the nucleus, which helps in the formation of viral replication complexes that can promote viral DNA replication, expression of late viral proteins, and ultimately lytic infection (70). Duck hepatitis B virus (DHBV) has been reported to rely on the recognition of RNA packaging signals by viral reverse transcriptase (RT), which can be efficiently activated by HSC70 and HSP40, thereby initiating downstream replication and nucleocapsid assembly (71). Enterovirus A71 (EV-71) is a positive-strand RNA virus in which the initiation of viral protein translation is guided by an internal ribosomal entry site (IRES), and HSC70 can upregulate the activity of IRES in cells to assist viral translation by interaction, thereby promoting the expression of viral proteins in RD cells (26). As mentioned before, IAV is a negative-sense single-stranded RNA virus that can utilize autophagy to facilitate its replication (72). Recent research has found that IAV induces autophagy through the binding of hemagglutinin (HA) to HSP90AA1 distributed on the cell surface. The interaction of HA1 and HSP90AA1 inhibits the phosphorylation of mTOR and AKT to induce autophagy through the AKT-MTOR pathway and thereby promote IAV replication (64).

In addition to protein-protein interactions, HSPs can also promote translation by binding to the viral genome. HSC70 can favor virus replication by binding regulator non-coding RNA (ncRNA). Studies have reported that many viruses, such as human immunodeficiency virus (HIV) (73), DENV (74), and West Nile virus (WNV) (75), encode microRNA-like ncRNA to regulate virus replication. Similarly, rabies virus (RABV) transcribes a small ncRNA, called leader RNA (leRNA). It was also found that HSC70 binds to leRNA to regulate viral replication during infection. Hepatitis C virus (HCV) is currently causing a worldwide epidemic. The nonstructural (NS) proteins are responsible for replication of HCV RNA as well as viral particle assembly, and are primary antiviral targets (76). In a recent study, Li et al. found that HSC70 co-precipitates with HCV NS proteins and RNA, interacting with the HCV replication complex and participating in HCV replication by regulating RNA translation from the HCV genome (77).

## Viral folding, encapsidation and assembly

After completion of translation in the cell, the virus usually recruits several host factors to facilitate assembly and budding. Immunogold labeling revealed that HSC70 is attached to the surface of HCV particles by interacting with the HPD (His-Pro-Arg) motif on the E2 envelope protein of the virus. Then HSC70, HCV core, and E2 proteins were found to co-localize at the periphery of lipid droplets, an important site for HCV assembly and release (78). By using an allosteric HSC70 inhibitor and RNAi-mediated knockdown, Khachatoorian et al. (79)demonstrated that inactivation of HSC70 reduces the speed of HCV particle assembly, thus concluding that HSC70 plays a role in the assembly of viral particles during HCV infection. HSP90 was also found to play an important role in the maturation of viral proteins, including helping viral particle assembly, protein folding, and maintaining protein activity. HSP90 was found to be involved in multiple viral activities, including capsid precursor processing in coxsackieviruses, polioviruses and rhinoviruses (80), viral capsid assembly in early hepatitis E viral infection (81), maintenance of L protein stability in lacrosse virus (28), maintenance of reverse transcriptase activity in hepatitis B virus and NS2/3 protease in hepatitis C virus, as well as assistance in viral L polymerase folding in measles and Nipah virus (82).

In the context of the global coronavirus pandemic, research on HSPs and their roles in coronavirus infection is very popular. Here, we summarize the findings on the role of HSP90 in coronavirus (CoV) maturation. After CoVs invade cells, large numbers of proteins are translated in the endoplasmic reticulum (ER), which causes ER stress and triggers the unfolded protein response (UPR). HSP90 regulates the UPR by stabilizing the ER stress sensor transmembrane kinase IRE1α, which in turn contributes to viral protein folding and replication (27). In this regard, a recent analysis of RNA-sequencing data from COVID-19 patients also suggested that inhibition of HSP90 could reduce the replication rate of the novel coronavirus (preprint data) (83). This idea has been confirmed in numerous reports of HSP90 inhibitor experiments, which found that that HSP90 inhibitors such as 17-AAG and Luminespib trigger the activities of the unfolded protein response (UPR) in mice, which protected endothelial cells in the pulmonary aorta and pulmonary microvasculature (84). According to recent studies on coronaviruses, HSP90 is considered to be a host-dependent factor for human coronaviruses MERS-CoV, SARS- CoV and SARS-CoV-2. Li et al. found that the depletion of Hsp90β, the cytosolic isoform of HSP90, profoundly reduced viral growth as shown by both viral load quantification and virion titration (85). As confirmed by co-immunoprecipitation, MERS-CoV nucleocapsid protein (NP) is a substrate of HSP90β, which maintains the stability of NP by directly binding it and thereby preventing its degradation by the proteasome. Similarly, they also conducted experiments on the proliferation process of SARS-CoV and SARS-CoV-2, which revealed that the inhibition of HSP90 leads to a significant reduction of virion production. HSP70 and HSP90 are of great importance for viral gene expression since they play a key role in assembling the capsid of some viruses. Viruses utilize HSP70 and HSP90 to fold their proteins and increase their chances of survival under unfavorable host conditions (86).

## Development of antiviral drugs targeting heat shock proteins

In many studies on various viruses, the heat shock protein family has been shown to be extensively involved in the viral life cycle, and there have been many advances in the development of antiviral drugs targeting the heat shock protein family. Antiviral drugs targeting heat shock proteins work in three general ways, either by inhibiting the ATPase activity of HSPs, inhibiting the ability of HSPs to form complexes, or triggering modifications of HSPs such as phosphorylation and acetylation to reduce their activity (87). Hsp90 is thought to be the most abundant and evolutionarily conserved heat shock protein. There is also a wealth of research on HSP90-targeted drugs such as geldanamycin (GM) (88), tanespimycin (17-AAG) (89) and histone deacetylase inhibitors (90). Hsp90 inhibitors were demonstrated to protect cultured cells against infection by EV-A71 (91). Similarly, HSP70 is active in various phases of infection by HCV, Flavivirus and Enterovirus, while HSP70 inhibitors such as quercetin, VER155008 and JC40 also show great potential in the treatment of these viruses (92–94). In the treatment of COVID-19, the clinically approved HSP60 inhibitor mizoribine was found to exert an antiviral effect and is considered to be a potentially beneficial agent for hypertensive patients infected with the new coronavirus (95, 96). Quercetin is an inhibitor of HSP70 that also inhibits the activity of HSP40, and was found to decrease the intracellular accumulation of infectious particles when applied in the treatment of HCV infection (97). Among small heat shock proteins, HSP27 has been studied more frequently, and 1,3,5-trihydroxy-13,13-dimethyl-2H-pyran [7,6-b] xanthone (TDP), a compound isolated from a traditional Chinese herb, was found to inhibit HSP27 with significant anti-cytopathic effects, leading to the inhibition of EV-A71 infection (98, 99).

## Summary

HSP family members participate in the promotion or inhibition of viral infection in many different ways. HSPs inhibit viral infection by acting on different client proteins, not only by activating immune pathways and regulating the cell cycle, but also by directly binding to proliferation-related factors of viruses to silence their replication. However, viruses also often hijack these molecular chaperones, the HSP family members are also extensively involved in all phases of viral proliferation (**Table 2**). The powerful regulatory ability of HSPs originates from numerous client proteins and more researches are required to explore the detailed mechanisms by which HSPs fight against viruses and help viral infections. In the process of viral infection, HSPs play different roles according to the different clients they serve, which makes them target proteins for the treatment of viral infections. Nowadays there are effective inhibitors, but few of them have become clinically approved drugs for complex reasons such as cell toxicity, side effects and drug stability.

**Table 2:** The involvement of heat shock proteins in various stages of viral infection.

Viral life cycle steps	HSPs species	Functions	References
Binding and internalization	HSP60 HSP70 HSP90	Involved in clathrin-mediated endocytosis Directly binds to viral molecules as a receptor on the cell surface	Howe et al.,2016 (61) Chuang et al.,2015 (58) Speth et al,1999 (100)
Viral uncoating and transport	HSP40 HSP70 HSP100	Associates with viral capsid proteins and facilitates virion assembly Drives membrane translocation during viral entry	Taguwa et al.,2015 (93) Ravindran et al.,2015 (101)
Genome replication and viral polyprotein translation	HSP40 HSP60 HSP70 HSP90	Enhances nuclear import of the viral ribonucleoprotein (vRNP) complex Facilitates vRNP stabilization Viral RNA synthesis Enhances viral polymerase activity	Batra et al.,2016 (25) Cao et al.,2014 (102) Naito et al.,2007 (103) Park et al.,2002 (104)
Encapsidation and assembly	HSP70 HSP90	Virion assembly	Gurer et al.,2005 (105) Radhakrishnan et al.,2010 (106)
Virion morphogenesis and budding	HSP60 HSP70 HSP90	Viral protein folding Viral protein stabilization	Zhang et al.,2005 (107) Katoh et al.,2017 (108)

In the background of the current global challenge of COVID-19, the development of drugs targeting heat shock proteins will be a new challenge and focus in this field. Therefore, a summary of the mechanisms of heat shock proteins in viral infection and the development of related inhibitor drugs offers a theoretical basis for future scientific exploration.

## Author contributions

XZ: writing-original draft. WY: conceptualization, funding acquisition and writing-review and editing. All authors contributed to the article and approved the submitted version.

## Funding

This work was financially supported by the National Natural Science Foundation of China (No. 31972623).

## Conflict of interest

The authors declare that the research was conducted in the absence of any commercial or financial relationships that could be construed as a potential conflict of interest.

## Publisher’s note

All claims expressed in this article are solely those of the authors and do not necessarily represent those of their affiliated organizations, or those of the publisher, the editors and the reviewers. Any product that may be evaluated in this article, or claim that may be made by its manufacturer, is not guaranteed or endorsed by the publisher.
